# Use of Model-Based Compartmental Analysis and a Super-Child Design to Study Whole-Body Retinol Kinetics and Vitamin A Total Body Stores in Children from 3 Lower-Income Countries

**DOI:** 10.1093/jn/nxz225

**Published:** 2019-09-18

**Authors:** Jennifer Lynn Ford, Joanne Balmer Green, Marjorie J Haskell, Shaikh M Ahmad, Dora Inés Mazariegos Cordero, Anthony Oxley, Reina Engle-Stone, Georg Lietz, Michael H Green

**Affiliations:** 1 Department of Nutritional Sciences, College of Health and Human Development, The Pennsylvania State University, University Park, PA, USA; 2 Program in International and Community Nutrition, Department of Nutrition, University of California, Davis, Davis, CA, USA; 3 International Centre for Diarrhoeal Disease Research, Dhaka, Bangladesh; 4 Laboratorio de Bioquímica Nutricional, Unidad de Nutrición y Micronutrientes, Instituto de Nutrición de Centroamérica y Panamá, Guatemala City, Guatemala; 5 Human Nutrition Research Centre, Newcastle University, Newcastle Upon Tyne, United Kingdom

**Keywords:** children, model-based compartmental analysis, retinol, stable isotopes, super-child design, tracer kinetics, vitamin A assessment, vitamin A stores, WinSAAM

## Abstract

**Background:**

Model-based compartmental analysis has been used to describe and quantify whole-body vitamin A metabolism and estimate total body stores (TBS) in animals and humans.

**Objectives:**

We applied compartmental modeling and a super-child design to estimate retinol kinetic parameters and TBS for young children in Bangladesh, Guatemala, and the Philippines.

**Methods:**

Children ingested [^13^C_10_]retinyl acetate and 1 or 2 blood samples were collected from each child from 6 h to 28 d after dosing. Temporal data for fraction of dose in plasma [^13^C_10_]retinol were modeled using WinSAAM software and a 6-component model with vitamin A intake included as weighted data.

**Results:**

Model-predicted TBS was 198, 533, and 1062 μmol for the Bangladeshi (age, 9–17 mo), Filipino (12–18 mo), and Guatemalan children (35–65 mo). Retinol kinetics were similar for Filipino and Guatemalan groups and generally faster for Bangladeshi children, although fractional transfer of plasma retinol to a larger exchangeable storage pool was the same for the 3 groups. Recycling to plasma from that pool was ∼2.5 times faster in the Bangladeshi children compared with the other groups and the recycling number was 2–3 times greater. Differences in kinetics between groups are likely related to differences in vitamin A stores and intakes (geometric means: 352, 727, and 764 μg retinol activity equivalents/d for the Bangladeshi, Filipino, and Guatemalan children, respectively).

**Conclusions:**

By collecting 1 or 2 blood samples from each child to generate a composite plasma tracer data set with a minimum of 5 children/time, group TBS and retinol kinetics can be estimated in children by compartmental analysis; inclusion of vitamin A intake data increases confidence in model predictions. The super-child modeling approach is an effective technique for comparing vitamin A status among children from different populations. These trials were registered at www.clinicaltrials.gov as NCT03000543 (Bangladesh), NCT03345147 (Guatemala), and NCT03030339 (Philippines).

## Introduction

Over the past 35 y, application of model-based compartmental analysis to retinol kinetic data has led to important advances in our understanding of whole-body vitamin A metabolism and homeostasis in rats [for reviews, see (1, 2)]. The approach has also been used to describe and quantify retinol kinetics and vitamin A total body stores (TBS), an indicator of vitamin A status (3), in humans [e.g., see (4, 5) and for reviews, see (2, 6, 7)]. However, to date, only a few modeling studies have been done in children (8). Thus, current knowledge is limited regarding potential differences in vitamin A kinetics in children compared with adults [as is the case in rats (9)] and regarding possible developmental changes in vitamin A metabolism and TBS.

The main limitation to applying traditional modeling approaches in children is that frequent and repeated blood sampling is required to adequately define retinol kinetics over time. Thus, some researchers have used population-based (“super-child”) approaches that require collection of only a few blood samples from each child; data from all children are combined into a composite (super-child) data set for analysis (10). A population-based approach has been used in several previous vitamin A studies in young children (8, 11, 12), including 2 experiments designed to determine group mean vitamin A kinetics and TBS in Mexican children aged 1–3 y (8) and 5–6 y (12). In both cases, TBS was also estimated for individual children using a retinol isotope dilution (RID) equation that included model-predicted values for the coefficients (*Fa* and *S*) used in the prediction equation to account for absorption, retention, and mixing of the tracer dose (13, 14). In addition, a super-child modeling approach was recently tested using theoretical data and shown to provide accurate prediction of population TBS and adequate estimation of retinol kinetic parameters for the group (15). The approach also provided accurate prediction of group-specific values for RID coefficients that, when used in the specified RID equation, resulted in good predictions of TBS in individuals.

The super-child approach has also been implemented in more recent studies designed to evaluate vitamin A status in children in Bangladesh, Guatemala, and the Philippines as part of the multisite Global Vitamin A Safety Assessment (GloVitAS) project. Viewed together, these studies provide extensive retinol kinetic data to which model-based compartmental analysis could be applied. Here, we present comparative results for retinol kinetics and TBS for children studied in these 3 countries based on population modeling of composite vitamin A tracer kinetic data sets. Modeling was also used to calculate group-specific values for RID equation coefficients that will be useful in predicting TBS in individual subjects. Our results show that population modeling, with a reduced sampling burden on each subject, is a feasible approach for studying retinol kinetics and estimating TBS in children.

## Methods

### Subjects

For the GloVitAS studies, children were recruited in Bangladesh, Guatemala, and the Philippines; see **Supplemental Methods** for more details. In brief, anthropometric measurements obtained for all subjects at baseline included length or height and body weight; participant age was recorded; hemoglobin and C-reactive protein were measured in a capillary blood sample during screening; and vitamin A intakes were assessed. Written consent was obtained from mothers of all children and procedures were approved by the appropriate ethical review boards. In this paper, we focus on results related to the application of compartmental analysis to predict parameters describing whole-body vitamin A kinetics and TBS, as well as RID equation coefficients, for the 3 groups. Additional details related to methodologies, demographics, and TBS for individual subjects, as well as information about other measured variables that are beyond the scope of this paper, will be presented in related papers that are currently in preparation [Ahmad et al. (Bangladesh); Mazariegos Cordero et al. (Guatemala), and Haskell, Engle-Stone et al. (the Philippines)].

As summarized here and in Supplemental Methods, subjects in the 3 groups differed in age, dietary vitamin A intake, and participation in high-dose supplementation programs. Briefly, for the study in Bangladesh, children included in the current analysis (9–17 mo; *n* = 87) had recently received a high-dose vitamin A supplement as part of a national vitamin A supplementation program and were consuming primarily breast milk; estimated vitamin A intakes ranged from ∼200 to 900 μg retinol activity equivalents (RAE)/d. For the study in Guatemala, vitamin A intakes in the children included here (35–65 mo; *n* = 135) were ∼150–2200 μg RAE/d. Children had not received high-dose vitamin A supplements in the previous 6 mo. Included Filipino children (12–18 mo; *n* = 120) had received their most recent high-dose vitamin A capsule in the previous 1–6 mo and were consuming ∼100–4300 μg RAE/d.

### Study design

On day 0, all children received an oral dose of 400 μg (1.17 μmol) [8-,9-,10-,11-,12-,13-,14-,15-,19-,20–^13^C_10_]retinyl acetate (98.8% pure; Buchem BV) in 0.2 mL sunflower oil via direct displacement pipet; Bangladeshi children were then breastfed, Filipino children were either breastfed or provided a cracker with butter and jam, and Guatemalan children consumed a breakfast meal (fried black bean paste, scrambled eggs cooked with vegetable oil, white bread, and orange juice). All mothers were instructed to provide their child's usual diet for the duration of the study.

The original intention for the GloVitAS studies was to implement the following super-child design at all sites: there was to be a minimum of 50 children/group and 2 blood samples would be obtained from each child between 6 h and 28 d after isotope administration; all children would be sampled at 4 d and each child would be sampled at 1 additional, randomly assigned time (6, 9, or 12 h, and 1, 2, 7, 11, 16, 22, or 28 d). This protocol was used for the studies in Guatemala and the Philippines, but in the case of Bangladesh, the design was modified to accommodate other aspects of the study that are not included in the current analysis. Specifically, for Bangladeshi subjects, 1 blood sample was collected from each child during the 28 d experiment; 40 children were sampled at 4 d, 5–6 children were sampled at each of the remaining 10 times, and 4 of the children were sampled twice, at 22 and 28 d.

### Sample procedures and retinol analyses

Venous blood (2–6 mL) was collected into tubes containing EDTA and tubes were transported on cold packs to the laboratory. Samples were centrifuged and resultant plasma was stored at –80°C (Bangladesh and the Philippines) or –20°C (Guatemala) until shipment on dry ice to Newcastle University for retinol analyses. Samples were protected from light during processing, storage, and shipment.

As described in Supplemental Methods, retinol was extracted from plasma using a modification of the method of Aebischer et al. (16) and liquid chromatographic conditions were adapted from Kane and Napoli (17). Selected reaction monitoring and quantification of [^12^C]retinol, [^13^C_10_]retinol, and [^12^C]retinyl acetate (internal standard) were done as previously described (18).

### Super-child data sets

For each study, fraction of the dose (FD_p_) of [^13^C_10_]retinol in plasma was calculated for each child at each sampling time as {[^13^C_10_]retinol (μmol/L) × estimated plasma volume (L)}/dose (μmol), where plasma volume was estimated with use of the regression equation of Linderkamp et al. (19) (see Supplemental Methods). Composite (population) super-child data sets were generated for modeling by calculating the geometric mean of the actual sampling times and FD_p_ at each time. Plasma retinol pool size (μmol) was calculated for each child at each sampling time as plasma retinol concentration (μmol/L) × estimated plasma volume, where retinol concentration was calculated as μmol/L [^13^C]retinol + μmol/L [^12^C]retinol. Then, geometric mean plasma retinol pool size was calculated based on all samples in each study and used in the steady state model solution (see next section).

### Kinetic analysis

**FIGURE 1:**
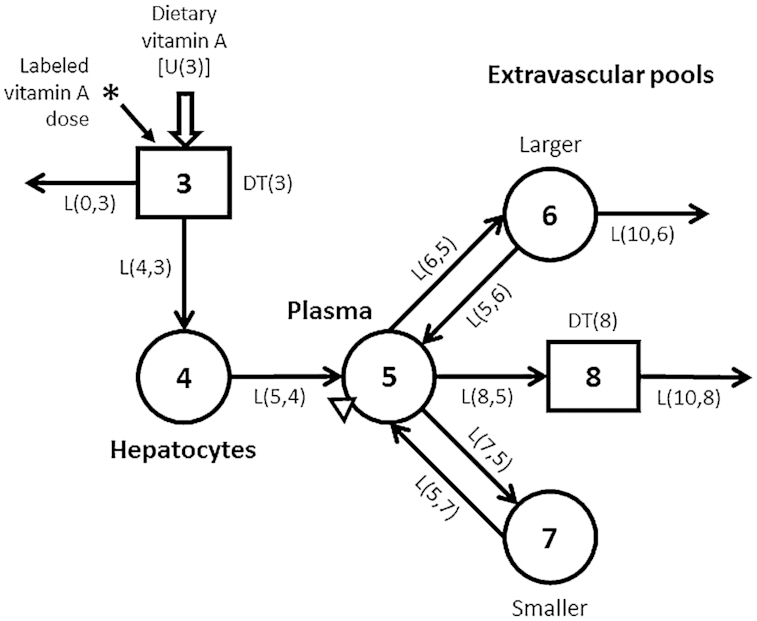
Proposed compartmental model for vitamin A kinetics in children. Circles represent compartments; the rectangles are delay elements; interconnectivities between components (arrows) are fractional transfer coefficients [L(I, J)s, or the fraction of retinol in compartment J transferred to compartment I each day]; and DT(I)s are delay times (or the time spent in delay element I). Delay element 3 is the site of introduction of ingested tracer (*) and dietary vitamin A [U(3)]. Components 3 and 4 represent vitamin A digestion and absorption plus chylomicron processing until vitamin A uptake by hepatocytes (compartment 4). Retinol bound to retinol-binding protein is secreted from compartment 4 into plasma compartment 5, the site of sampling (triangle). Retinol in plasma can exchange with vitamin A in 2 extravascular pools (a larger compartment 6 and a smaller compartment 7) and it can also irreversibly enter component 8, hypothesized to be tissues from which vitamin A does not recycle. Compartment 6 and component 8 are the sites of irreversible loss from the system.

For each study, data for geometric mean FD_p_ were plotted versus time (6 h to 28 d) to generate composite (super-child) isotope response curves. Then, model-based compartmental analysis was applied using the Windows version of the Simulation, Analysis and Modeling software [WinSAAM version 3.3.0 (20–22)] in light of a 6-component model describing whole-body vitamin A kinetics in children (**Figure 1**). The model shown in Figure 1 and described in the figure legend was derived from a previous model (8), but includes updated hypotheses that are justified in more detail in Supplemental Methods. Briefly, in addition to irreversible loss from the larger storage pool (compartment 6), the current model includes an additional site of output after retinol in plasma compartment 5 is transferred, for functional use or nonfunctional disposal, to tissues from which there is no retinol recycling (delay component 8). This pathway has been included in 1 previously published model for vitamin A metabolism in humans (23), but not in others (4, 8). Here, we added it to reflect the likely transfer of absorbed retinol and its use by tissues from which there is no recycling, thus accounting for the portion of ingested vitamin A that is absorbed but not retained (24–26). We used a delay component (rather than a compartment) to reflect the assumption that there would be a finite amount of time [DT(8) = 75 min; see Supplemental Methods] between tissue uptake of retinol and its catabolism; and we partitioned the rate of loss from tissues so that 50% of the output was from tissue component 8 and 50% was from storage compartment 6. For the former, the value for L(8,5) was calculated as follows and fixed in the model for each group: assuming a steady state and 80% absorption efficiency (24, 27), vitamin A disposal rate (DR; μmol/d) was estimated for each data set as geometric mean adjusted vitamin A intake (see next paragraph) [U(3)] × 0.8; then, the value calculated for 50% of the DR was divided by the plasma retinol pool size [M(5), μmol] to calculate L(8,5) (d ^−1^).

Once a satisfactory fit was obtained between observed data and model predictions by manual curve fitting, weighted nonlinear regression analysis was performed in WinSAAM to estimate final values for model parameters. Specifically, for each super-child data set, geometric mean FD_p_ data were weighted using a fractional standard deviation (FSD) of 0.05 at all times, except at 4 d when the weight was increased to 0.025 because of the larger number of samples at that time. Geometric mean adjusted vitamin A intake for included children from each country was added in the model as weighted input, using an FSD of 0.05 (28). Because assessed intake included both preformed vitamin A and provitamin A, the estimated value for provitamin A carotenoids (as RAE) was adjusted to the equivalent amount of preformed vitamin A (see Supplemental Methods for details). Final values for parameters provided by nonlinear regression included delay times [DT(I)s, or the time spent in delay component I] and fractional transfer coefficients [L(I, J)s, or the fraction of retinol in compartment J transferred to compartment I each day] and their statistical uncertainties (FSDs). Then, geometric mean plasma retinol pool size was fixed and, using a steady state solution, compartment masses [M(I)], including vitamin A masses in compartments 6 and 7 (TBS), and transfer rates [R(I, J)], including DR, were calculated in WinSAAM. Other parameters such as days of vitamin A stores, system fractional catabolic rate, transit times, residence times, and recycling number and time were also calculated; see Cifelli et al. (2) for additional details.

### Determination of coefficients for use in RID equations

We also used the super-child model to calculate group-specific values for the coefficients *Fa* and *S* that are included in RID equations such as the one presented by Green et al. (14), which can be used to predict vitamin A TBS in individuals. In the equation TBS = *Fa* × *S* × 1/SA_p_ (14), *Fa* is the fraction of the oral tracer dose absorbed and retained in stores (compartments 6 and 7; Figure 1) at time *t*; it was calculated from the model as [F(6)_t_ + F(7)_t_], where F(I) is the fraction of an orally administered tracer dose in compartment I as a function of time. *S* is retinol specific activity in plasma/stores at time *t* and was calculated as [F(5)_t_/ M(5)] / {[F(6)_t_ + F(7)_t_] / [M(6) + M(7)]}.

### Data manipulations and statistics

**FIGURE 2:**
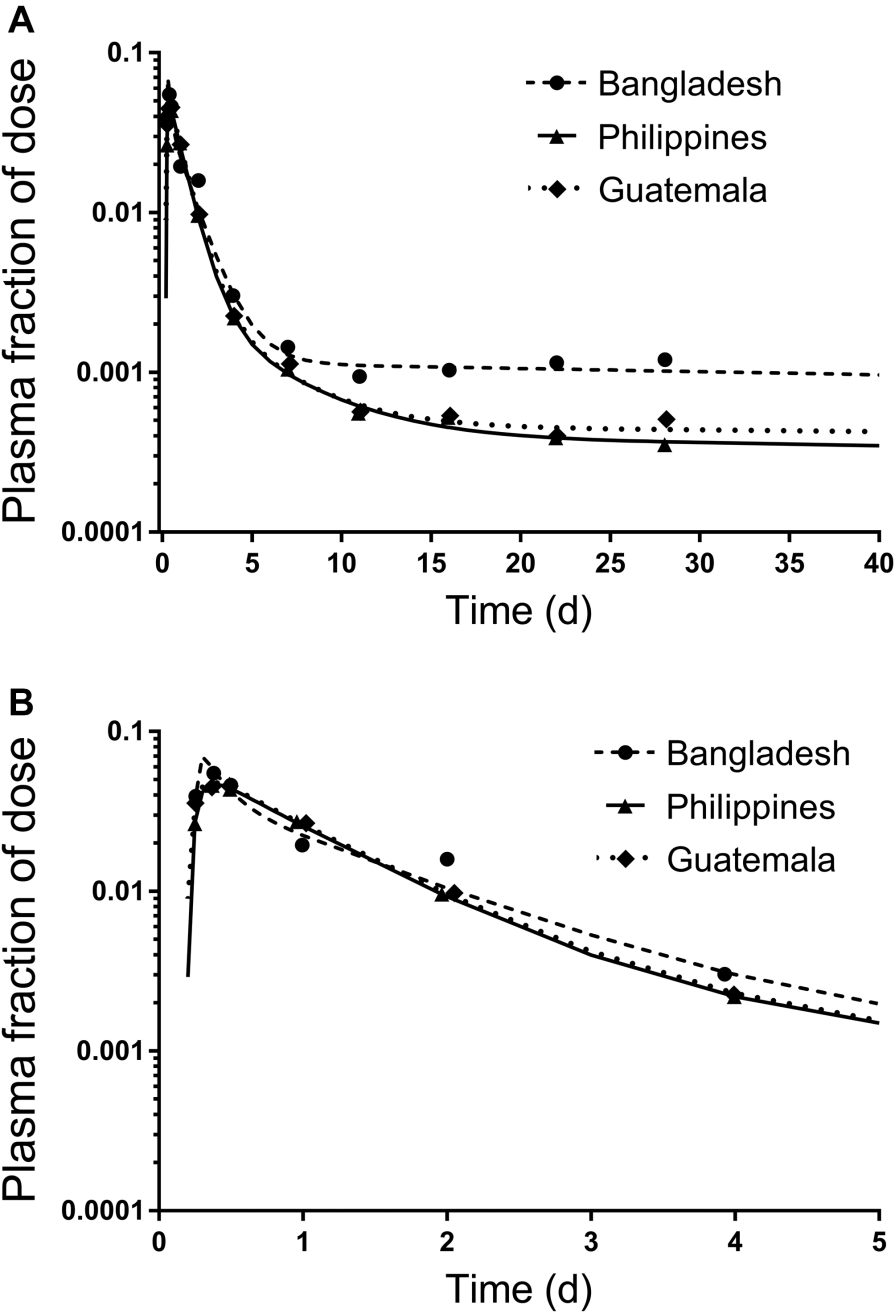
Geometric mean observed and model-predicted fraction of dose for [^13^C_10_]retinol in plasma simulated to 40 d (A) or 5 d (B) for Bangladeshi, Guatemalan, and Filipino children. Symbols are observed data and lines are model simulations. For the Bangladeshi group, the super-child data set included 5–6 children/time except at 4 d when there were 40 children; for the Filipino group, there were 9–13 children/time and 112 at 4 d; the Guatemalan group included 8–16 children/time and 135 at 4 d. Mean data for each group were fit independently using the model shown in Figure 1. The model-calculated weighted sums of squares for plasma were 6.2E-07, 1.9E-08, and 5.6E-08 for the Bangladeshi, Filipino, and Guatemalan groups, respectively.

**FIGURE 3:**
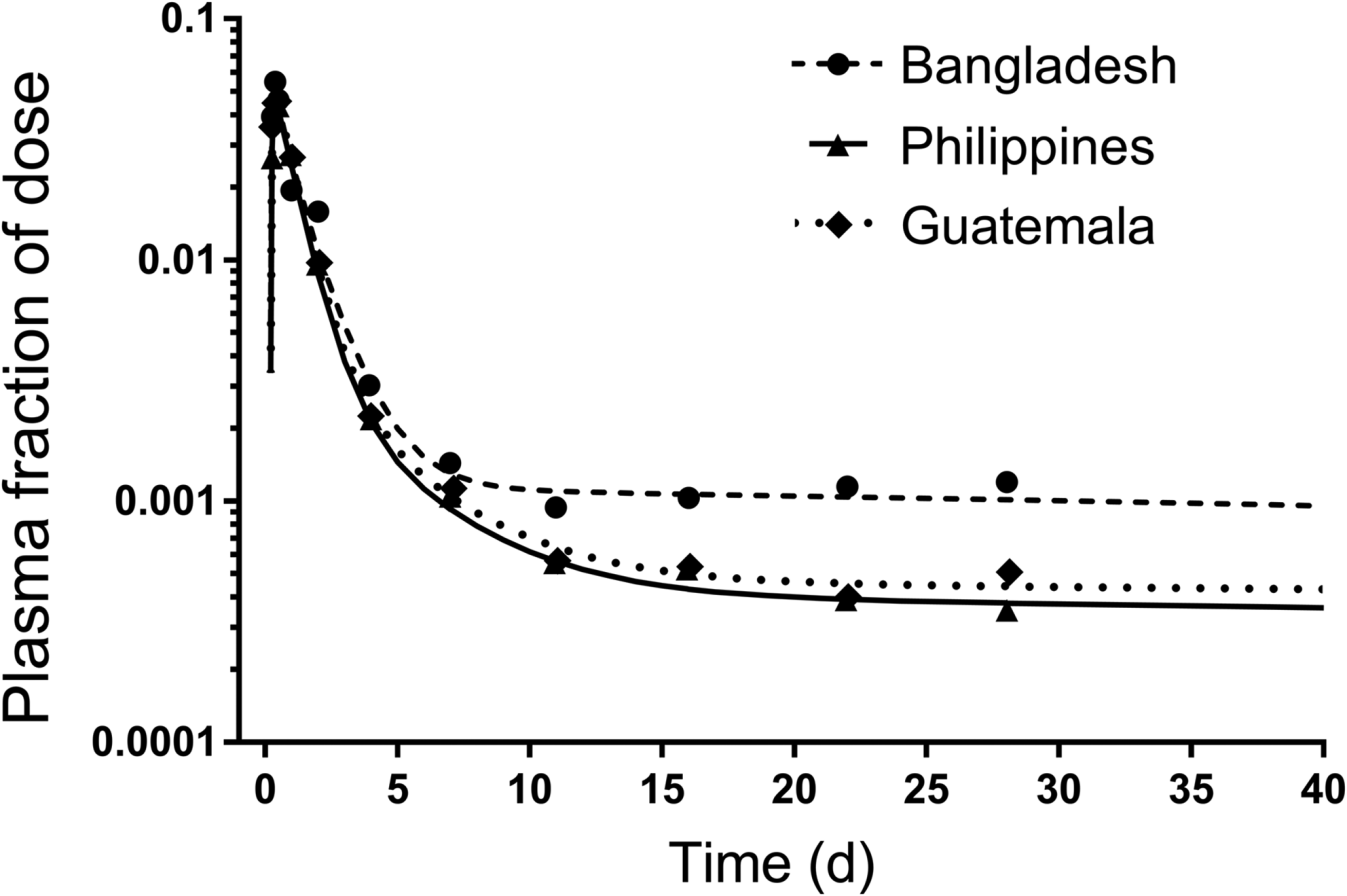
Geometric mean observed and model-predicted fraction of dose for [^13^C_10_]retinol in plasma versus time for Bangladeshi, Guatemalan, and Filipino children. Symbols are observed data and lines are model simulations using the partially parallel model (see Results). For the Bangladeshi group, the super-child data set included 5–6 children/time except at 4 d when there were 40 children; for the Filipino group, there were 9–13 children/time and 112 at 4 d; the Guatemalan group included 8–16 children/time and 135 at 4 d. Mean data for the 3 groups were fit with use of a partially parallel modeling approach (see Methods for details); the model is shown in Figure 1. The model-calculated weighted sums of squares for plasma were 2.4E-07, 4.8E-08, and 8.8E-08 for the Bangladeshi, Filipino, and Guatemalan groups, respectively.

Data were managed in Microsoft Excel. Graphics were created using Microsoft PowerPoint and Adobe Photoshop (Figure 1) and GraphPad Prism 7.0 for Windows (**Figures 2** and **3**). For modeling, parameter identifiability was evaluated based on values for parameter FSDs calculated using WinSAAM (20).

## Results

### Group characteristics

Group mean ages were 12, 14, and 50 mo for the Bangladeshi, Filipino, and Guatemalan children, respectively, and corresponding mean body weights were 8.4, 8.8, and 15 kg. Group mean plasma retinol concentrations were 0.785, 1.02, and 1.24 μmol/L, and geometric mean vitamin A intakes based on dietary assessment were 352, 727, and 764 μg RAE/d, respectively.

### Kinetic data and modeling analysis

For modeling, each super-child data set (i.e., observed geometric mean data for FD_p_ versus time) was fit using the model shown in Figure 1, with all model parameters adjustable except that L(4,3) was fixed at 0.8 and L(0,3) at 0.2 to reflect an absorption efficiency of 80%, DT(8) was fixed at 75 min, and L(8,5) was fixed at the value calculated for each group to reflect 50% of the DR leaving the system via the nonexchangeable tissue pool (component 8). Values for vitamin A intake used as weighted modeling input, adjusted to include provitamin A carotenoids, were 1.24, 2.56, and 2.83 μmol RAE/d (356, 733, and 810 μg RAE/d) for the Bangladeshi, Filipino, and Guatemalan groups.

Observed data to 28 d and model predictions simulated to 40 d are presented in Figure 2 for the 3 groups and corresponding model results are presented in **Supplemental Table 1**. The steady state model solution predicted a TBS (vitamin A in compartments 6 plus 7) of 199 μmol for the Bangladeshi children, 535 μmol for the Filipino children, and 1068 μmol for the Guatemalan children (Supplemental Table 1). Visual inspection of Figure 2A indicates that the model provided a good fit to the data for each group. Curves for the Filipino and Guatemalan groups were very similar. However, when observed data were plotted as geometric mean plasma retinol specific activity (fraction of dose/μmol) versus time (data not shown), the plasma retinol response profile for the Guatemalan group was consistently lower than that for the Filipino group because plasma retinol pool size was larger for the former. Overall, for these groups, the lower the plasma retinol specific activity, the higher the TBS.

As shown in Figure 2B, early data (e.g., from 6 h to 4 d), reflecting the kinetics of vitamin A absorption and early tracer distribution, were surprisingly similar among groups. In addition, similarities in the model predictions for parameters were observed, particularly between the Filipino and Guatemalan groups (see Supplemental Table 1). These observations led us to hypothesize that the data sets could be combined and fit using a parallel or partially parallel model. For this alternative modeling approach, we first set values for all adjustable parameters to be equal among groups, except that, as in the initial analyses, L(4,3) (Figure 1) was fixed at 0.8, L(0,3) at 0.2, L(8,5) at the calculated value for each group, and DT(8) was 75 min. Because model predictions were not compatible with the observed plasma tracer data for all groups, we systematically changed specific adjustable parameters to be independent (i.e., not equal), as needed. We found that, for the model to accurately predict the value for dietary intake used as weighted input and to obtain 50% loss from the larger exchangeable storage compartment 6, the fractional loss from that compartment [L(10,6)] needed to be independent among groups. After detailed examination, differences were noted in tracer redistribution from and recycling to plasma for the Bangladeshi group compared to the others, and thus values for L(5,4), L(7,5), and L(5,7) were made independent for that group. In addition, there was more extensive recycling in Bangladeshi children, who had lower vitamin A intakes and stores compared to the other groups, and thus the value for L(5,6) was also made independent. In summary, for the final partially parallel model, one adjustable parameter [L(10,6)] was independent among groups; in addition, L(5,4), L(7,5), L(5,7), and L(5,6) were independent for the Bangladeshi group; the remaining adjustable parameters were set equal for the 3 groups (see **Supplemental WinSAAM Deck**). As shown in Figure 3, the final model fits to the 3 data sets using the partially parallel model are indistinguishable from those obtained when each group's super-child data set was modeled independently (Figure 2A). Use of the partially parallel model (compared with fitting the data sets independently) provided a more robust fit to the data by increasing the degrees of freedom (from 0.57 to 1.5); also model parameters were estimated with more confidence and parameter identifiability was improved (**Supplemental Table 2**); thus, results are presented for this approach.

Final kinetic parameters (fractional transfer coefficients and delay times) determined using the partially parallel model are shown in **Table 1** and their statistical uncertainties are presented in Supplemental Table 2. The model predicted that it took 5 h [DT(3)] for retinol absorption, incorporation into chylomicrons, and chylomicron metabolism until uptake of chylomicron remnants by hepatocytes (compartment 4; Figure 1). For the Bangladeshi children, whose dietary vitamin A intakes were lower, hepatocyte chylomicron-derived retinol was mobilized into plasma bound to retinol-binding protein ∼2 times faster than in the other groups. Similarly, in the Bangladeshi group, 28 pools of plasma retinol were turned over each day, compared with ∼17 pools for the Filipino and Guatemalan groups, with 50% and <20%, respectively, of that plasma turnover transferred to the smaller exchangeable pool (compartment 7). Thus, for the Filipino and Guatemalan children, larger portions of plasma retinol turnover (70% and 74%) were transferred to the larger exchangeable pool (compartment 6; presumably retinyl esters). For all groups, <13% of retinol left plasma irreversibly via nonexchangeable tissues (component 8). In addition, 159% of the smaller exchangeable pool recycled to plasma each day for the Bangladeshi group compared to 34% for the Filipino and Guatemalan groups, whereas ∼2% and 0.9%, respectively, of the larger exchangeable pool recycled each day. Although differences were subtle, the fraction of the larger pool that was irreversibly lost each day increased as vitamin A stores decreased.

**TABLE 1:** Model-derived retinol kinetic parameters for 3 groups of young children^1^

Parameter	Bangladesh	Philippines	Guatemala
DT(3), d	0.206	0.206	0.206
L(4,3), d^−1^	0.8	0.8	0.8
L(0,3), d^−1^	0.2	0.2	0.2
L(5,4), d^−1^	2.15	1.22	1.22
L(7,5), d^−1^	13.9	3.07	3.07
L(5,7), d^−1^	1.59	0.340	0.340
L(6,5), d^−1^	12.3	12.3	12.3
L(5,6), d^−1^	0.0217	0.00884	0.00884
L(10,6), d^−1^	0.00246	0.00194	0.00108
L(8,5), d^−1^	1.30	2.21	1.34
DT(8), d	0.052	0.052	0.052
L(10,8), d^−1^	1	1	1

1 Values are model-predicted fractional transfer coefficients [L(I, J)s, or the fraction of retinol in compartment J transferred to compartment I each day] and delay times [DT(I)s, or delay time spent in compartment I] determined for geometric mean data sets for Bangladeshi, Filipino, and Guatemalan children using the model shown in Figure 1. For the Filipino and Guatemalan groups, results were calculated using a partially parallel model in which values for the following parameters were set equal between groups: DT(3), L(5,4), L(7,5), L(5,7), L(6,5), and L(5,6). For the Bangladeshi group, values for DT(3) and L(6,5) were set equal to those in the other 2 groups but values for L(5,4), L(5,6), L(7,5), and L(5,7) were independent and adjustable in the model. For all 3 groups, L(10,6) was adjustable and independent. In addition, L(8,5) was fixed for each group at the value calculated to be 50% of the disposal rate/M(5) (see Methods) and DT(8) was fixed at 75 min. See Supplemental Table 2 for corresponding statistical uncertainties for parameters.

Steady state model predictions are presented in **Table 2**. Results showed that, for the Bangladeshi children, the mass of retinol in hepatocytes [M(4)] was about 25% of that predicted for the Filipino and Guatemalan children, presumably reflecting the lower vitamin A intake for the former group. Plasma retinol pool [M(5)] was ∼2 times larger in the Guatemalan children compared with the 2 younger groups because this pool's size is dependent on age and body weight. For all groups, the mass of vitamin A in compartment 7 was 0.7–1.7% of that in compartment 6. Vitamin A in the larger extravascular pool was lowest (195 μmol) in the Bangladeshi children; it was 2.7 and 5.4 times higher in the Filipino and Guatemalan children, respectively; nearly all of TBS (198, 533, and 1062 μmol, respectively) was accounted for by this pool.

**TABLE 2:** Steady state compartment masses, transfer rates, and other model-derived parameters for 3 groups of young children^1^

Parameter	Bangladesh	Philippines	Guatemala
Compartment masses, μmol			
M(4)	0.453	1.67	1.85
M(5)	0.382	0.462	0.848
M(6)	195	529	1054
M(7)	3.34	4.17	7.65
TBS	198	533	1062
Transfer rates, μmol/d			
U(3)	1.22	2.56	2.83
R(5,4)	0.974	2.04	2.26
R(7,5)	5.31	1.42	2.60
R(5,7)	5.31	1.42	2.60
R(6,5)	4.70	5.68	10.4
R(5,6)	4.23	4.68	9.32
R(10,6)	0.480	1.03	1.14
R(8,5)	0.497	1.02	1.14
Absorbed and retained, %	76.2	69.9	73.6
Disposal rate, μmol/d	0.976	2.05	2.27
Days of stores, d	203	260	467
System fractional catabolic rate, d^−1^	0.00492	0.00384	0.00214

1 Values are compartment masses [M(I), or μmol of vitamin A in compartment I (see Figure 1)] and transfer rates [R(I, J), or the rate of transfer of retinol in compartment J to compartment I each day] calculated using a steady state solution in WinSAAM; TBS = M(6) + M(7) and disposal rate = R(10,6) + R(8,5); the % of ingested vitamin A that was absorbed and retained in stores was calculated as {[R(6,5) + R(7,5)]/[R(6,5) + R(7,5) + R(8,5)] × 0.8} × 100, based on the assumption that 80% of the dose was absorbed; days of vitamin A stores were calculated as TBS/disposal rate and the system fractional catabolic rate was calculated as vitamin A disposal rate/TBS. TBS, total body stores.

Also shown in Table 2 are vitamin A transfer rates between compartments. Results indicate that both the rate of plasma turnover to the larger exchangeable storage pool and recycling from that pool to plasma were ∼2 times higher in the Guatemalan children compared to the other groups. For all groups, the % of the dose that was absorbed and retained, assuming 80% absorption efficiency, ranged from 70% to 76%. Vitamin A DR was ∼2 times higher in the Filipino and Guatemalan children compared to the Bangladeshi children; this finding is directly related to differences in dietary intake across groups. The model predicted that the Bangladeshi children had 6.7 mo of vitamin A stores compared with 8.5 mo for the Filipino children and 15.4 mo for the Guatemalan children; the same trend is seen for the system fractional catabolic rate, which is the inverse of days of stores.

Additional calculated and time-related kinetic parameters for the groups are shown in **Table 3**. For the Bangladeshi children, transit time in both plasma and the larger exchangeable pool was ∼2 times faster than for the Filipino and Guatemalan children and transit time in the smaller exchangeable pool was ∼5 times faster. In addition, the number of times retinol recycled back to plasma was highest in the Bangladeshi children (10, compared to 3 in the Filipino children and 5 in the Guatemalan children). Recycling time was similar to transit time in the larger exchangeable pool for the Filipino and Guatemalan children and half that for the Bangladeshi children. Overall, the system residence time was highest for the Guatemalan children (469 d) at more than ∼2 times higher than for the others.

**TABLE 3:** Additional calculated and time-related kinetic parameters^1^

Parameter	Bangladesh	Philippines	Guatemala
Mean transit time			
$\quad \bar{t}$(5), h	0.873	1.36	1.44
$\quad \bar{t}$(6), d	41.4	92.8	101
$\quad \bar{t}$(7), d	0.629	2.94	2.94
Residence time			
$\bar{T}$(5,5), d	0.392	0.226	0.374
$\quad \bar{T}$(6,5), d	200	258	465
$\quad \bar{T}$(7,5), d	3.42	2.04	3.37
$\quad \bar{T}$(SYS), d	204	260	469
Plasma recycling number [$\nu $(5)]	9.77	2.97	5.25
Plasma recycling time [$\overline {tt} $(5), d]	20.8	87.5	89.2

1 Values are mean transit times [$\bar{t}$(7), or the mean of the distribution of times that retinol entering compartment I spends there before leaving reversibly or irreversibly]; residence times [$\bar{T}$(I,5), or the mean of the distribution of times that retinol spends in compartment I after reaching plasma compartment 5]; plasma recycling number, or the average number of times retinol recycles to plasma before leaving plasma irreversibly, and recycling time, or the time required for an average retinol molecule leaving plasma to cycle back to plasma. For more details on calculations, see reference 2.

### Model-predicted RID equation coefficients

We used the final super-child modeling results obtained using the partially parallel model to calculate values for the composite coefficient (*FaS*) for use in an RID equation. Values for *FaS* at 4 d were 1.58 for the Bangladeshi group, 2.44 for the Filipino group, and 2.93 for the Guatemalan group; values calculated for each day from 4 to 28 d are presented in **Supplemental Table 3**.

## Discussion

In this work, we applied compartmental modeling and a population-based (super-child) approach to estimate whole-body retinol kinetics, TBS, and group-specific RID coefficients in groups of young Bangladeshi, Filipino, and Guatemalan children as part of the GloVitAS project. Model-predicted total body vitamin A stores were 198, 533, and 1062 μmol, respectively, for the 3 groups (aged 9–17, 12–18, and 35–65 mo), presumably reflecting differences in assessed vitamin A intakes (352, 727, and 764 μg RAE/d) and high-dose vitamin A supplementation. Our analyses demonstrate the usefulness of the super-child modeling approach for quantifying vitamin A kinetics in children, in whom frequent and repeated blood sampling is generally not feasible. For the GloVitAS studies, 2 blood samples were collected from most subjects and then composite tracer response curves were analyzed; similar population-based approaches are recommended in pediatric pharmacokinetics (29) and have been used previously in the vitamin A field (8, 11, 12). Based on our current work, we recommend several changes in the experimental design used for future super-child studies; as detailed in the **Supplemental Discussion**, these include a sample size of 60 children/group, with all children sampled at 7 d for estimation of TBS by RID, and a study length of 5 h to 42 d, with 13 sampling times.

Although vitamin A intake data were collected as part of each GloVitAS study, we did not originally see this information as key to the modeling aspects of the project. However, in light of recent work (28, 30), we included an estimate of vitamin A intake as weighted input during modeling. Although intake data have been included in 2 other modeling studies (8, 30), and their usefulness has been confirmed by Green's laboratory using theoretical data (28), the current work represents the first multisite vitamin A modeling studies that include vitamin A intake as weighted data. The additional data constrain the model and lead to more accurate definition of the terminal slope of the plasma isotope response curves, a factor required for accurate prediction of TBS, especially if modeling reveals that studies were not of optimal duration. This appears to be the case for the Filipino and Guatemalan children, for whom composite tracer response data (Figures 2A and 3) did not reach a terminal slope by 28 d. In consideration of these factors, we conclude that inclusion of vitamin A intake data results in increased confidence in model predictions. In fact, when vitamin A intake was not included in the model, the predicted terminal slope was much steeper for the Filipino and Guatemalan groups. This resulted in TBS predictions that were only 21% of the values predicted when intake was included; also model-predicted intakes were 260% and 410% higher, respectively, than the adjusted intake used as weighted data. In the case of the Bangladeshi group, when intake was not included, the terminal slope converged to zero, resulting in a 36% higher predicted TBS, and model-predicted intake was only 14% of the adjusted intake compared to when intake data were included. Addition of intake data also prevented the terminal slope from converging to zero in our work with Lopez-Teros et al. (8). As we have previously shown using theoretical data (28), addition of a reasonable estimate of intake (±50% of the actual value) is sufficient. Thus, we recommend including vitamin A intake data in future modeling studies.

Another new aspect of this work is that, in contrast to previously published models of vitamin A metabolism (4, 8), the current model (Figure 1) includes system output from a nonexchangeable tissue pool (delay component 8) as well as from the large extravascular storage pool (compartment 6). Both component 8 and compartment 6 comprise extravascular tissues that take up retinol from plasma (compartment 5); however, once retinol enters component 8, it is destined for irreversible utilization, whereas it can either be used or recycled to plasma from compartment 6. We hypothesize that the pathway from plasma to component 8 reflects the likelihood that some of the retinol taken up from plasma by tissues (31) is catabolized to retinoic acid or other metabolites. This pathway represents the portion of the incoming labeled vitamin A dose that is “absorbed but not retained” as proposed in earlier work (24–26).

The original super-child modeling protocol designed for the GloVitAS studies was based on a model with only 1 extravascular compartment (a large storage compartment representing TBS); sensitivity analysis (32) was used to determine the minimum number and timing of blood samples. We found that the current data sets required a model with 2 exchangeable extravascular compartments but, because of the inclusion of vitamin A intake as weighted data and with fixing the value for L(8,5), the sampling protocol was still sufficient to identify the model structure. Application of a partially parallel model, in which some parameters were set equal among groups, resulted in better identification of model parameters (Supplemental Table 2) as well as similar agreement between model predictions and the observed data to that when the groups were modeled individually (Figures 2A and 3). In addition, for all groups, predictions of TBS were within ∼1% using the 2 modeling approaches (Table 2 and Supplemental Table 1).

Although there is limited published information on vitamin A kinetics in children, some useful comparisons can be made between the current results and data published by Lopez-Teros et al. in 2017 (8). In that study of young Mexican children, retinol kinetics were even faster than those determined here for the Bangladeshi children and much faster than those observed for the Guatemalan and Filipino children. For example, secretion of diet-derived retinol from hepatocytes [L(5,4), Figure 1] was ∼10 times faster in the Mexican subjects and ∼2 times faster in the Bangladeshi children than in the Filipino and Guatemalan children. In addition, the Mexican and Bangladeshi children had a smaller mass of retinol in hepatocyte compartment 4 (∼6% and 25% of the value predicted for the other 2 groups). These differences likely reflect the lower assessed vitamin A intakes by the Mexican and Bangladeshi children compared with those of the Filipino and Guatemalan children (on average, <400 μg RAE/d compared with >700 μg RAE/d) and may suggest saturation of hepatocyte retinol-binding protein during times of high vitamin A intake. For the former groups, retinol turnover from plasma was faster (28 and 85 pools/d) compared to the latter (∼17 pools/d), with a larger portion of this turnover transferred to the smaller exchangeable pool (compartment 7) compared to the larger compartment 6. Moreover, recycling of retinol from tissues to plasma was higher in the Mexican and Bangladeshi groups (65 and 10, respectively, compared with 3–5 for the Filipino and Guatemalan groups); also, fractional recycling from compartment 6, as well as from compartment 7, to plasma was faster (∼2%/d for the Mexican and Bangladeshi children and 0.09%/d for the other 2 groups). Taken together, these results suggest that lower vitamin A intakes, such as in the Mexican and Bangladeshi children, may increase retinol recycling and mobilization of vitamin A stores to maintain plasma retinol homeostasis.

Some interesting ideas emerge by comparing TBS in light of vitamin A intakes among the 3 groups studied here and Mexican children aged 3–6 y (TBS, 862 μmol) (12) and 1–3 y (TBS, 844 μmol) (8). First, assuming vitamin A intakes were relatively similar for the 2 groups of Mexicans, results for TBS suggest that children may have reached a balanced state by age 1–3 y and that vitamin A stores were maintained by adequate and balanced intakes. In contrast, the surprisingly similar vitamin A kinetics but different TBS in the Filipino (12–18 mo; 533 μmol) and Guatemalan children (35–65 mo; 1062 μmol) studied here may suggest a different response. Specifically, because high vitamin A intake can lead to positive vitamin A balance, one might hypothesize that if the Filipino children continue to consume high amounts of vitamin A, in 2–4 y, TBS might reach or surpass the value predicted for the Guatemalan children.

In addition to estimating TBS and retinol kinetics for groups in the current studies, we also used the model to calculate group-specific values for the time-variant RID equation coefficient *FaS* (Supplemental Table 3). Values at 4 d were 1.58 (Bangladeshi children), 2.44 (Filipino children), and 2.93 (Guatemalan children); these will be used, along with plasma retinol specific activity at 4 d, to estimate TBS in individual children in the detailed publications about each study. Current values for *FaS* at 4 d are similar to those reported by Lopez-Teros et al. in Mexican children aged 1–3 y (8) and 3–6 y (12) (1.85 and 2.30, respectively). As more studies are completed, it will be worth comparing values for the composite coefficient across populations and at different times to determine whether there is a universal time and *FaS* value that could be used in various populations.

In summary, our results demonstrate the power of applying compartmental analysis to super-child data sets to obtain information on group retinol kinetic parameters, TBS, and the composite RID coefficient. Although TBS can be estimated in individual children using RID after collection of only 1 blood sample, investigators can obtain additional useful information on population retinol kinetics and a more accurate estimate of the RID coefficient *FaS* by collecting 1 other sample as part of a super-child design. Such information is important in assessing the impact of intervention programs on vitamin A status in children.

## Supplementary Material

nxz225_Supplemental_File

## Notes

Support for this work was provided by the Bill & Melinda Gates Foundation (Project Number OPP1115464).

Author disclosures: JLF, JBG, MJH, SMA, DIMC, AO, RE-S, GL, and MHG, no conflicts of interest.

Supplemental Methods, Supplemental Tables 1–3, Supplemental Discussion, Supplemental Figure 1, Supplemental WinSAAM Deck, and Supplemental References are available from the “Online Supporting Material” link in the online posting of the article and from the same link in the online table of contents at http://jn.nutrition.org.

JLF and MHG contributed equally to this work.

Data supporting this publication are openly available at https://doi.org/10.25405/data.ncl.9771902.v1.

Abbreviations used: DR, disposal rate; DT, delay time; FD_p_, fraction of administered dose in plasma; FSD, fractional standard deviation; GloVitAS, Global Vitamin A Safety Assessment; RAE, retinol activity equivalent(s); RID, retinol isotope dilution; TBS, total body store(s); WinSAAM, Windows version of the Simulation, Analysis and Modeling software.
