# Supplementary material for: Use of Model-Based Compartmental Analysis and a Super-Child Design to Study Whole-Body Retinol Kinetics and Vitamin A Total Body Stores in Children from 3 Lower-Income Countries
Source: J Nutr. 2019 Sep 18;150(2):411–8. doi: 10.1093/jn/nxz225 (PMC7004890; doi:10.1093/jn/nxz225)
Supplement: nxz225_Supplemental_File [file nxz225_supplemental_file.pdf]

Supplemental Methods

Subjects. Bangladeshi infants were recruited from the urban slum areas of Kamrangir Char and Hazaribagh in Dhaka; study procedures were approved by the Institutional Review Board of the International Centre for Diarrhoeal Disease Research, Bangladesh (icddr,b). In Guatemala, subjects were recruited from several urban centers (Santa Catarina Pinula and La Comunidad, Mixco) and from 2 semi-urban communities (San Miguel Dueñas and Santa María de Jesus); procedures were approved by the Institutional Ethics Committee of INCAP. Filipino children were recruited from low-income neighborhoods in Mandaluyong City within Metro-Manila; the study protocol was approved by the Institutional Review Board at the University of California, Davis and by the Research Ethics Board of the University of the Philippines, Manila. All studies were also approved by the Research Ethics Board of Newcastle University.

Assessment of vitamin A intake. Vitamin A intake was estimated for the Bangladeshi children based on measured breastmilk vitamin A concentrations and published estimates of breastmilk intake (Supplemental References 1,2) and by doing 24 h dietary recalls on 2 separate days and a 30 d food frequency questionnaire. Estimates for dietary intake were determined using a food composition table from the Institute of Nutrition and Food Science at the University of Dhaka and included both preformed vitamin A and provitamin A carotenoids [as RAE based on the IOM conversion factors (Supplemental Reference 3)]. For the Guatemalan children, dietary vitamin A intake was assessed by using a 30 d food frequency questionnaire as well as 24 h recalls done on 3 separate days during the study; the latter included weighing mother-reported portions of consumed foods. Estimated intake, based on the INCAP Food Composition Table, included both preformed vitamin A and provitamin A carotenoids [as RAE using the IOM conversion factors (Supplemental Reference 3)]. For the Filipino children, dietary vitamin A intake was assessed by conducting 24 h recalls on 4 – 5 separate days during the study, a 12 h in-home observation to weigh and record the child's food intake (with 12 h recall to complete the 24 h period), and a 30 d questionnaire to assess use of vitamin A-containing supplements. For breastfeeding children, breast milk intake was measured using the $^{2}H_2O$ dose-to-mother method (Supplemental References 4,5). Results were combined to estimate usual vitamin A intake using the US National Cancer Institute method (Supplemental Reference 6); the estimate included preformed vitamin A and provitamin A carotenoids [as RAE based on the IOM conversion factors (Supplemental Reference 3)].

Calculation of vitamin A intake for modeling. Because assessed vitamin A intake included both preformed and provitamin A, we adjusted the value for provitamin A carotenoids (as RAE) to the equivalent amount of preformed vitamin A. That is, because the conversion of provitamin A carotenoids to RAE [using the IOM conversion factors (Supplemental Reference 3)] includes absorption and conversion efficiency, and we assumed an absorption efficiency of 80% for preformed vitamin A for modeling, the value for vitamin A intake used as weighted input in the model was calculated as: preformed vitamin A + (provitamin A / 0.8). More specifically, for the Bangladeshi and Guatemalan children, the individual assessed preformed and provitamin A intakes were used in the above calculation; the geometric mean of the individual adjusted estimates was used in the model. For the Filipino children, the geometric mean value for assessed preformed and provitamin A intake was used in the above calculation. Preformed vitamin A contributed, on average, >95% of total vitamin A intake for the Bangladeshi subjects, >90% for the Filipinos, and ~76% for the Guatemalan children.

Retinol analyses. Retinol was extracted from plasma using a modification of the method of Aebischer et al. (16). Briefly, 400 µL of plasma was diluted with 400 µL diH_20 before addition of 800 µL ethanol containing 50 pmol [^{12}C]retinyl acetate as internal standard. The denatured sample was extracted twice with 2 mL of hexane and hexane in the combined extracts was evaporated under N_2. The residue was resuspended in 100 µL of ethanol and 10 µL was used for LC-MS/MS analysis. Liquid chromatographic conditions were adapted from Kane and Napoli (17) to a Shimadzu Prominence HPLC equipped with a 100 mm x 2.1 mm 3 µm Supelcosil ABZ PLUS column (Supelco) and a 4 x 2 mm SecurityGuard (Phenomenex) C_{18} cartridge maintained at 25°C. The binary mobile phase system consisted of 0.1% formic acid aqueous (A) and 0.1% formic acid in acetonitrile (B); separation of analytes was done at a flow rate of 400 µL/min and a linear gradient of 60% B to 95% B from 0.0 – 6.0 min; 100% B from 6.1 – 13.0 min; 60% B from 13.0 – 14.0 min; and then 60% B from 14.0 – 17.0 min to re-equilibrate the column. The HPLC was coupled to an API 4000 MS/MS system (AB SCIEX) with atmospheric pressure chemical ionization in positive ion mode. Optimized atmospheric pressure chemical ionization source parameters were: CUR 20 psig; GS1 40 psig; GS2 40 psig; CAD 6 psig; TEM 300°C; NC 5 µA. Selected reaction monitoring and quantitation of [^{12}C]retinol, [^{12}C]retinyl acetate, and [$^{13}C_{10}$]retinol was done as previously described (18).

Plasma volume estimation. Calculation of fraction of dose in plasma required an estimate of plasma volume for each child. For this work, we estimated plasma volume using the regression equation of Linderkamp et al. (19). Specifically, blood volume (L) was estimated based on body weight (kg) and height (cm); then, hemoglobin concentration was used to estimate hematocrit as hemoglobin × 3 (Supplemental Reference 7), and plasma volume was estimated as blood volume × (1 – hematocrit).

Rationale for updated hypotheses in the compartmental model. The model shown in Figure 1 was adapted from a previously-published model for vitamin A kinetics in children (8). Specifically, in addition to irreversible loss from the larger storage compartment 6, the current model includes an additional site of loss from extrahepatic tissues that do not recycle retinol to plasma (component 8). The hypothesis that 2 tissue sites of irreversible loss are required was supported by modeling tracer data for plasma and loss in rats [unpublished observations, MH Green, based on data presented in (Supplemental Reference 8)] in which, to adequately fit the loss data, the model required output from both an exchangeable and a nonexchangeable extravascular compartment (analogous to compartment 6 and component 8, respectively). In that analysis, a delay of 75 min was required to fit the loss data. Thus, we fixed the delay time in component 8 [DT(8)] at 75 min for all 3 groups in the current analysis. Note that the value assigned to DT(8) does not influence model results and was chosen to provide a more physiologically-sound model. Since further testing using theoretical data (unpublished observations, JL Ford and MH Green) indicated that, without loss data, one cannot accurately distinguish the proportion of output from these 2 sites, for the current work, we partitioned the rate of loss from tissues so that 50% of the output (i.e., disposal rate) was from tissue component 8 and 50% was from storage compartment 6.

Supplemental Discussion

Future recommendations for the super-child approach. Based on the current analysis, the following revised design is recommended for future super-child studies (**Supplemental Figure 1**). We recommend that there be a sample size of at least 60 children per group and that 2 blood samples be collected from each child at times (n=13) from 5 h to 42 d. Based on sensitivity analysis performed using WinSAAM (Supplemental Reference 9), the recommended sampling times are 5, 8, and 12 h, and 1, 2, 4, 7, 10, 14, 21, 28, 35, and 42 d. All children should be sampled at 7 d (see below) and at one additional time, randomly assigned at recruitment, such that 5 children will be sampled at each of the 12, non-7 d times.

Accuracy related to administration of the labeled oral dose is crucial. That is, the time and volume or, preferably, the weight of the dose administered needs to be precisely recorded at the time of dosing (d 0). The amount of label administered to each child must be adequate to ensure accurate detection 42 d later, based on detection capability of the equipment that will be used for analysis.

For the current modeling analysis, absorption efficiency for preformed vitamin A was assumed to be 80% (24,27). To improve the accuracy of predictions by the model, investigators should consider actually determining absorption of vitamin A. For example, the dual isotope absorption method (Supplemental Reference 10) can be used to measure vitamin A absorption by administering an oral dose of labeled vitamin A followed ~3 h later by an intravenous dose of a differently-labeled vitamin A in a physiological lipid emulsion; absorption can be calculated as the ratio of fraction of dose of orally ingested vitamin A / intravenously injected vitamin A at a time after the ratio in plasma has stabilized (e.g., 14 d in rats).

In addition to predicting a group mean value for TBS from modeling the composite dataset, data from the plasma samples collected on d 7 can be used in an RID equation to estimate TBS in individual children. Since in more recent work (15), we found that 7 d was a better time than 4 d for doing RID in children, we recommend the former time (7 d) for future studies. Specifically, the super-child model can be used to calculate time-variant values for the equation coefficients (*Fa* and *S*) to be applied in the equation along with each child's retinol specific activity in plasma (SA_p) at the corresponding time (d 7). Note that values for the coefficients can be calculated at all times and values from 4 to 42 d can be used in the equation to predict TBS for individuals sampled at those times. It is important to mention that, while we hypothesize that 7 d will be more accurate than 4 d for estimating the absolute value for TBS in individual children by RID when a group model-predicted value for *FaS* is used, TBS predictions for the group should be approximately the same at both times. In addition, use of 4 d data can still be valuable for ranking subjects within a group from low to high TBS and estimating the distribution and range of TBS for the group.

Supplemental Table 1 Group-independent model-derived retinol kinetic parameters and steady state compartment masses for 3 groups of young children[1]

	Bangladesh	Philippines	Guatemala
Parameter			
DT(3), d	0.240	0.207	0.191
L(4,3), d^{-1}	0.8	0.8	0.8
L(0,3), d^{-1}	0.2	0.2	0.2
L(5,4), d^{-1}	6.37	1.17	1.17
L(7,5), d^{-1}	42.8	2.74	2.47
L(5,7), d^{-1}	3.31	0.270	0.316
L(6,5), d^{-1}	12.8	11.5	12.0
L(5,6), d^{-1}	0.0227	0.00806	0.00856
L(10,6), d^{-1}	0.00248	0.00194	0.00106
L(8,5), d^{-1}	1.30	2.21	1.34
DT(8), d	0.052	0.052	0.052
L(10,8), d^{-1}	1	1	1
Compartment masses			
M(4), µmol	0.154	1.74	1.94
M(5), µmol	0.382	0.462	0.848
M(6), µmol	194	530	1061
M(7), µmol	4.94	4.71	6.63
TBS, µmol	199	535	1068

[1]Values are model-predicted parameters and steady state compartment masses calculated by the model when applied independently to geometric mean datasets for Bangladeshi, Filipino, and Guatemalan children. Parameters include fractional transfer coefficients [L(I,J)s, or the fraction of retinol in compartment J transferred to compartment I each day] and delay times [DT(I)s, or delay time spent in compartment I]. Compartment masses [M(I), or µmol of vitamin A in compartment I] were calculated using the steady state model solution in WinSAAM. See Supplemental Table 2 for statistical uncertainties for these parameters. The model is shown in Figure 1. TBS, total body stores.

Supplemental Table 2 Fractional standard deviations for group-independent versus partially-parallel model-derived kinetic parameters for 3 groups of young children[1]

	Bangladesh		Philippines		Guatemala	
Parameter	Independent	Parallel	Independent	Parallel	Independent	Parallel
DT(3)	0.033	0.013	0.019	0.013	0.031	0.013
L(5,4)	0.36	0.12	0.048	0.039	0.057	0.039
L(7,5)	0.30	0.27	0.13	0.10	0.18	0.10
L(5,7)	0.26	0.20	0.14	0.094	0.17	0.094
L(6,5)	0.027	0.015	0.028	0.015	0.027	0.015
L(5,6)	0.035	0.030	0.057	0.032	0.051	0.032
L(10,6)	0.12	0.12	0.13	0.13	0.12	0.12

[1]Shown are statistical uncertainties (fractional standard deviations) for adjustable kinetic parameters when super-child datasets for the 3 groups of young children were fit independently versus using a partially-parallel model. Parameters are delay times [DT(I)s, or delay time spent in compartment I] and fractional transfer coefficients [L(I,J)s, or the fraction of retinol in compartment J transferred to compartment I each day]. The model is shown in Figure 1.

Supplemental Table 3 Population-specific values for the composite coefficient (*FaS*) at various times for use in a retinol isotope dilution equation[1]

Time (d)	Bangladesh	Philippines	Guatemala
4	1.58	2.44	2.93
5	1.04	1.67	2.03
6	0.788	1.29	1.58
7	0.673	1.06	1.31
8	0.618	0.909	1.12
9	0.592	0.796	0.984
10	0.578	0.712	0.882
11	0.571	0.649	0.806
12	0.566	0.601	0.748
13	0.562	0.564	0.704
14	0.559	0.536	0.670
15	0.556	0.515	0.644
16	0.554	0.498	0.624
17	0.551	0.485	0.609
18	0.549	0.475	0.597
19	0.546	0.467	0.588
20	0.544	0.461	0.581
21	0.542	0.456	0.575
22	0.539	0.452	0.570
23	0.537	0.448	0.567
24	0.534	0.445	0.564
25	0.532	0.442	0.561
26	0.530	0.440	0.559
27	0.527	0.438	0.557
28	0.525	0.436	0.555

[1]Shown are model-calculated values for the time-variant composite coefficient *FaS* that can be applied in a retinol isotope diluatio equation such as Equation *1* to estimate TBS for individual children in each group. Values are calculated as $[F(6)_t + F(7)_t] \times \{F(5)_t/M(5) / [F(6)_t + F(7)_t / M(6)+M(7)]\}$, where F(I) is the fraction of tracer in compartment I as a function of time and M(I) is the mass of vitamin A in compartment I. The model is shown in Figure 1.

[^{13}C]Retinyl acetate (arrow at 5 h) **Retinol isotope dilution** (arrow at 7 d)

Child ID	5 h	8 h	12 h	1 d	2 d	4 d	7 d	10 d	14 d	21 d	28 d	35 d	42 d
1-5	✓						✓						
6-10		✓					✓						
11-15			✓				✓						
16-20				✓			✓						
21-25					✓		✓						
26-30						✓	✓						
31-35							✓	✓					
36-40							✓		✓				
41-45							✓			✓			
46-50							✓				✓		
51-55							✓					✓	
56-60							✓						✓
# sampled	5	5	5	5	5	5	60	5	5	5	5	5	5

Supplemental Figure 1. Recommended design for future super-child studies of vitamin A kinetics and status. The labeled dose is administered on d 0 and data from d 7 are used to calculated vitamin A total body stores using a retinol isotope dilution equation. See Supplemental Discussion for further details about the recommended design.

Supplemental WinSAAM Deck

```
A SAAM31    P-B-G GM ALL U(3) PARTIALLY-PARALLEL    [29-JUN-19]
CC GEOMETRIC MEAN PLASMA FRACTION OF DOSE VERSUS TIME FOR 3 GROUPS
CC INCLUDING BANGLADESHI, FILIPINO AND GUATEMALAN CHILDREN
CC COMPOSITE DATASETS COMBINED AND FIT USING PARTIALLY-PARALLEL MODEL
CC 6-COMPONENT MODEL FOR VITAMIN A KINETICS IN CHILDREN
CC DIETARY INTAKE [U(3)] USED AS WEIGHTED INPUT
CC FOR FILIPINO AND GUATEMALAN GROUPS: VALUES FOR ALL ADJUSTABLE
CC PARAMETERS SET EQUAL EXCEPT THAT L(10,6) INDEPENDENT AND ADJUSTABLE
CC FOR BANGLADESHI GROUP: VALUES FOR L(5,4), L(7,5), L(5,7), L(5,6), L(10,6)
CC WERE INDEPENDENT AND ADJUSTABLE
CC L(8,5) CALCULATED FOR EACH GROUP AS [(DIETARY INTAKE*0.8)/2]/M(5) AND FIXED
CC PHILIPPINES (N=120)
CC PARAMETER
H PAR
CC          VALUE         LOWER LIMIT    UPPER LIMIT
CC IC(I)=INITIAL CONDITION (FRACTION OF DOSE) IN COMPARTMENT I AT TIME 0
   IC(3)=1
CC DN(I)=NUMBER OF ELEMENTS IN DELAY COMPONENT I
CC DT(I)=DELAY TIME IN COMPONENT I (DAY)
   DT(3)     2.057179E-01  0.000000E+00   1.000000E+02
   DN(3)    8
CC L(I,J)=FRACTION OF J TRANSFERRED TO I PER DAY (DAY^-1)
CC ASSUMING 80% ABSORPTION EFFICIENCY
   L(4,3)   0.8
CC L(0,3)=FRACTIONAL LOSS OF UNABSORBED TRACER
   L(0,3)    0.2
   L(5,4)    1.223622E+00  0.000000E+00   1.000000E+02
   L(7,5)    3.071890E+00  0.000000E+00   1.000000E+02
   L(5,7)    3.405374E-01  0.000000E+00   1.000000E+02
   L(6,5)    1.231655E+01  0.000000E+00   1.000000E+02
   L(5,6)    8.835580E-03  0.000000E+00   1.000000E+02
CC L(10,6)=FRACTIONAL CATABOLIC RATE OF COMPARTMENT 6;
CC COMPARTMENT 10 IS LOSS FROM THE SYSTEM
   L(10,6)   1.939394E-03  0.000000E+00   1.000000E+02
CC L(8,5)=[(GM DIETARY INTAKE*0.8)/2]/M(5)
CC L(8,5) WAS FIXED AT THE CALCULATED VALUE
   L(8,5)    2.214
CC DT(8) FIXED AT 75 MIN
   DT(8)    0.052
   DN(8)    8
   L(10,8)=1
CC STEADY STATE SOLUTION
CC U(I)=VITAMIN A INTAKE RATE (UMOL/D)
CC M(I)=MASS OF VITAMIN A IN COMPARTMENT I (UMOL)
H STE
   U(3)      2.561592E+00 0             100
CC M(5)=PLASMA RETINOL POOL
CC GM M(5) CALCULATED FOR ALL CHILDREN AT ALL TIMES
   M(5)     0.4625
CC MODEL-PREDICTED MASSES IN COMPARTMENTS 6 & 7
CC M(6)=529
CC M(7)=4.17
H DAT
CC DIETARY VITAMIN A INTAKE [U(3)] AS WEIGHTED INPUT
```

```
CC          TIME (D)      U(3)           FRACTIONAL STANDARD DEVIATION
100                                      FSD=0.05
CC GM ASSESSED INTAKE=704 UG PREFORMED VITAMIN A/D + 23 UG PROVITAMIN A RAE/D
CC WEIGHTED U(3)=704+(23/0.8)=733 UG RAE/D=2.56 UMOL RAE/D
   U(3)     0             2.56
CC GEOMETRIC MEAN PLASMA FRACTION OF ORAL [13C10]RETINYL ACETATE DOSE
CC INCLUDED 9-13 CHILDREN/TIME AND 112 CHILDREN AT 4 D
105                                      FSD=0.05
CC           TIME (D)       PLASMA FRACTION OF ORAL DOSE
            0             0
            0.24819662    0.026361583
            0.369220693   0.04586997
            0.493377025   0.043454498
            0.9580861     0.027169027
            1.963451096   0.009556731
CC INCREASE WEIGHT TO ACCOUNT FOR INCREASE NUMBER OF CHILDREN AT 4 D
105                                      FSD=0.025
            3.995176137   0.002181794
105                                      FSD=0.05
            6.993033117   0.001045432
            10.92628846   0.000553514
            15.93270898   0.000522024
            21.95402784   0.000388812
            28.03872142   0.000352375
CC SIMULATIONS IN COMPARTMENTS 5, 6 AND 7
105
            0
2           0.05                         20
2           1                            27
2           10                           10
106
            0
2           0.05                         20
2           1                            27
2           10                           10
107
            0
2           0.05                         20
2           1                            27
2           10                           10
CC BANGLADESH
CC PARAMETER
H PAR
   IC(13)=1
   DT(13)=DT(3)
   DN(13)   8
   L(14,13) 0.8
   L(0,13)   0.2
   L(15,14)  2.152658E+00  0.000000E+00   1.000000E+02
   L(17,15)  1.392411E+01  0.000000E+00   1.000000E+02
   L(15,17)  1.593562E+00  0.000000E+00   1.000000E+02
   L(16,15)=L(6,5)
   L(15,16)  2.168768E-02  0.000000E+00   1.000000E+02
   L(20,16)  2.462724E-03  0.000000E+00   1.000000E+02
CC L(18,15)=[(DIETARY INTAKE*0.8)/2]/M(15)
   L(18,15) 1.298
```

```
   DT(18)   0.052
   DN(18)   8
   L(20,18)=1
CC STEADY STATE
H STE
   U(13)     1.219523E+00 0             100
CC M(15)=PLASMA RETINOL POOL
CC GM M(15) CALCULATED FOR ALL CHILDREN AT ALL TIMES
   M(15)    0.382
CC MODEL-PREDICTED MASSES IN COMPARTMENTS 16 & 17
CC M(16)=195
CC M(17)=3.34
H DAT
CC DIETARY VITAMIN A INTAKE [U(13)] AS WEIGHTED INPUT
CC          TIME (D)      U(13)          FRACTIONAL STANDARD DEVIATION
100                                      FSD=0.05
CC GM ASSESSED INTAKE=352 UG RAE/D
CC U(13)=GM OF INDIVIDUAL CHILD ESTIMATES FOR PREFORMED VA+(PROVITAMIN A/0.8)
CC WEIGHTED U(13)=356 UG RAE/D=1.24 UMOL RAE/D
   U(13)    0             1.24
CC GEOMETRIC MEAN PLASMA FRACTION OF ORAL [13C10]RETINYL ACETATE DOSE
CC INCLUDED 5-6 CHILDREN/TIME AND 40 CHILDREN AT 4 D
115                                      FSD=0.05
CC           TIME (D)       PLASMA FRACTION OF ORAL DOSE
            0             0
            0.255070515   0.039304484
            0.379283989   0.054793611
            0.498899587   0.046096319
            0.993991584   0.019386832
            2.001794497   0.015871874
CC INCREASE WEIGHT TO ACCOUNT FOR INCREASE NUMBER OF CHILDREN AT 4 D
115                                      FSD=0.025
            3.927820427   0.003016679
115                                      FSD=0.05
            6.993672175   0.001439822
            10.99022327   0.00094098
            16.00226079   0.001032172
            22.00956505   0.001148056
            28.04110436   0.001200537
CC SIMULATIONS IN COMPARTMENTS 15, 16 AND 17
115
            0
2           0.05                         20
2           1                            27
2           10                           10
116
            0
2           0.05                         20
2           1                            27
2           10                           10
117
            0
2           0.05                         20
2           1                            27
2           10                           10
CC GUATEMALA
CC PARAMETER
```

```
H PAR
   IC(23)=1
   DT(23)=DT(3)
   DN(23)   8
   L(24,23) 0.8
   L(0,23)   0.2
   L(25,24)=L(5,4)
   L(27,25)=L(7,5)
   L(25,27)=L(5,7)
   L(26,25)=L(6,5)
   L(25,26)=L(5,6)
   L(30,26)  1.077104E-03  0.000000E+00   1.000000E+02
CC L(28,25)=[(DIETARY INTAKE*0.8)/2]/M(25)
   L(28,25) 1.335
   DT(28)   0.052
   DN(28)   8
   L(30,28) 1
CC STEADY STATE
H STE
   U(23)     2.833704E+00 0              100
CC M(25)=PLASMA RETINOL POOL
CC GM M(25) CALCULATED FOR ALL CHILDREN AT ALL TIMES
   M(25)    0.848
CC MODEL-PREDICTED MASSES IN COMPARTMENTS 26 & 27
CC M(26)=1054
CC M(27)=7.65
H DAT   100                                    FSD=0.05
CC GM ASSESSED INTAKE=764 UG RAE/D
CC U(23)=GM OF INDIVIDUAL CHILD ESTIMATES FOR PREFORMED VA+(PROVITAMIN A/0.8)
CC WEIGHTED U(23)=810 UG RAE/D=2.83 UMOL RAE/D
   U(23)    0             2.83
CC GEOMETRIC MEAN PLASMA FRACTION OF ORAL [13C10]RETINYL ACETATE DOSE
CC INCLUDED 8-16 CHILDREN/TIME AND 112 CHILDREN AT 4 D
125                                    FSD=0.05
            0             0
            0.253637423   0.035501178
            0.368441789   0.044641924
            0.491466246   0.045459892
            1.022452771   0.026679538
            2.050807582   0.00975629
CC INCREASE WEIGHT TO ACCOUNT FOR INCREASE NUMBER OF CHILDREN AT 4 D
125                                    FSD=0.025
            3.992746907   0.002251418
125                                    FSD=0.05
            7.116623055   0.001131295
            11.05266214   0.000565315
            16.06009991   0.000534231
            22.04308722   0.000400275
            28.13302869   0.000506924
CC SIMULATIONS IN COMPARTMENTS 25, 26 AND 27
125
            0
2           0.05                        20
2           1                           27
2           10                          10
126
            0
```

```
2           0.05                            20
2           1                               27
2           10                              10
127
            0
2           0.05                            20
2           1                               27
2           10                              10
CC FaS PHILIPPINES
135G(35)
XG(35)=(F(6)+F(7))*(F(5)/M(5))/
         ((F(6)+F(7))/(M(6)+M(7)))
            1
2           1                               28
CC FaS BANGLADESH
145G(45)
XG(45)=(F(16)+F(17))*(F(15)/M(15))/
          ((F(16)+F(17))/(M(16)+M(17)))
            1
2           1                               28
CC FaS GUATEMALA
155G(55)
XG(55)=(F(26)+F(27))*(F(25)/M(25))/
          ((F(26)+F(27))/(M(26)+M(27)))
            1
2           1                               28
100
   M(4)
   M(5)
   M(6)
   M(7)
   U(3)
   R(10,6)
   R(8,5)
   M(14)
   M(15)
   M(16)
   M(17)
   U(13)
   R(20,16)
   R(18,15)
   M(24)
   M(25)
   M(26)
   M(27)
   U(23)
   R(30,26)
   R(28,25)
```

Supplemental References

1. Islam MM, Peerson JM, Ahmed T, Dewey KG, Brown KH. Effects of varied energy density of complementary foods on breast-milk intakes and total energy consumption by healthy, breastfed Bangladeshi children. Am J Clin Nutr 2006;83:851–8.
2. Islam MM, Khatun M, Peerson JM, Ahmed T, Mollah MA, Dewey KG, Brown KH. Effects of energy density and feeding frequency of complementary foods on total daily energy intakes and consumption of breast milk by healthy breastfed Bangladeshi children. Am J Clin Nutr 2008;88:84–94.
3. Institute of Medicine. Vitamin A. Dietary reference intakes for Vitamin A, Vitamin K, Arsenic, Boron, Chromium, Cooper, Iodine, Iron, Manganese, Molybdenum, Nickel, Silicon, Vanadium, and Zinc. 2001.
4. Coward WA, Cole TJ, Sawyer MB, Prentice AM. Breast-milk intake measurement in mixed-fed infants by administration of deuterium oxide to their mothers. Hum Nutr Clin Nutr 1982;36:141–8.
5. International Atomic Energy Agency. Stable isotope technique to assess intake of human milk in breastfed infants. IAEA, Vienna; 2010.
6. Tooze JA, Kipnis V, Buckman DW, Carroll RJ, Freedman LS, Guenther PM, Krebs-Smith SM, Subar AF, Dodd KW. A mixed-effects model approach for estimating the distribution of usual intake of nutrients: the NCI method. Stat Med 2010;29:2857–68.
7. Bain BJ, Lewis SM, Bates I. Basic haematological techniques. Dacie and Lewis practical haematology 10th ed. Philadelphia (PA): Churchhill Livingstone Elsevier; 2006. p. 25–57.
8. Green MH, Green JB. Multicompartmental analysis of whole body retinol dynamics in vitamin A sufficient rats. Fed Proc, Fed 1987;46:1011 (Abstr. 4047).
9. Wastney ME, Patterson BH, Linares OA, Greif PC, Boston RC. Testing robustness: sensitivity, identifiability, and stability. In: Investigating biological systems using modeling: strategies and software. San Diego (CA): Academic Press; 1999. p. 307–26.
10. Green MH, Green JB. Use of a plasma dual isotope ratio method to measure vitamin A absorption. FASEB J 1997;11:A142.
